# Health effects of non-occupational exposure to oil extraction

**DOI:** 10.1186/s12940-016-0140-1

**Published:** 2016-04-26

**Authors:** Cristina O’Callaghan-Gordo, Martí Orta-Martínez, Manolis Kogevinas

**Affiliations:** ISGlobal, Centre for Research in Environmental Epidemiology (CREAL), Doctor Aiguader, 88, 08003 Barcelona, Spain; Universitat Pompeu Fabra (UPF), Doctor Aiguader, 88, 08003 Barcelona, Spain; CIBER Epidemiología y Salud Pública (CIBERESP), Av. Monforte de Lemos, 3-5, 28029 Madrid, Spain; International Institute of Social Studies, Erasmus University Rotterdam, Kortenaerkade 12, 2518 AX The Hague, The Netherlands; Institut de Ciència i Tecnologia Ambientals (ICTA), Universitat Autònoma de Barcelona, 08193 Bellaterra, Cerdanyola del Vallès, Barcelona, Spain; IMIM (Hospital del Mar Medical Research Institute), Dr. Aiguader, 88, 08003 Barcelona, Spain

**Keywords:** Oil extraction industry, Non-occupational exposures, Crude oil

## Abstract

Oil extraction may cause extensive environmental impact that can affect health of populations living in surrounding areas. Large populations are potentially exposed to oil extraction related contamination through residence in areas where oil extraction is conducted, especially in low and middle income countries (LMICs). Health effects among people residentially exposed to upstream oil industry contaminants have been poorly studied. Health effects of exposure to oil related contamination have been mainly studied among cleanup workers after oil spills from tankers or offshore platforms.

In this paper we aim to identify the type and extension of residential exposures related to oil extraction activities and to comment on the few health studies available. We estimated that 638 million persons in LMICs inhabit rural areas close to conventional oil reservoirs. It is relevant to specifically study people residentially exposed to upstream oil industry for the following reasons: First, persons are exposed during long periods of time to oil related contamination. Second, routes of exposure differ between workers and people living close to oil fields, who can be exposed by ingestion of contaminated waters/foods and by dermal contact with contaminated water and/or land during daily activities (e.g. bathing, agricultural activities, etc.). Third, individuals potentially more susceptible to the effect of oil related contamination and not normally occupationally exposed, such as infants, children, pregnant women, elderly or people with previous health conditions, are also exposed.

There are few papers studying the potential health effects of residential exposure to oil related contamination, and most of them share important limitations. There is a need for more research through the conduct of methodologically robust studies in exposed populations worldwide. Despite the difficulties in the conduct of studies in remote areas, novel approaches, such as measurement of individual exposure using biomarkers of exposure and effect, should be used. These studies should be promoted to understand the health risks associated to residential exposure to oil related contamination, support effective control policies to avoid such contamination and to sustain public health recommendations and policies to avoid exposure in already contaminated areas.

## Background

Oil extraction may cause extensive environmental contamination and this may affect the health of population living in surrounding areas [1]. The health effects of exposure to oil related contamination have been mainly studied after oil spills among cleanup workers and residents of the affected coastal areas [2]. By contrast, the health effects among people residentially exposed to oil extraction related contamination (usually occurring in low-middle income countries -LMICs-) have been poorly studied. In this paper we identify the type and extension of residential exposures, comment on the few health studies available and identify this type of exposure as a priority for research and control.

## Main text

**Fig. 1:**
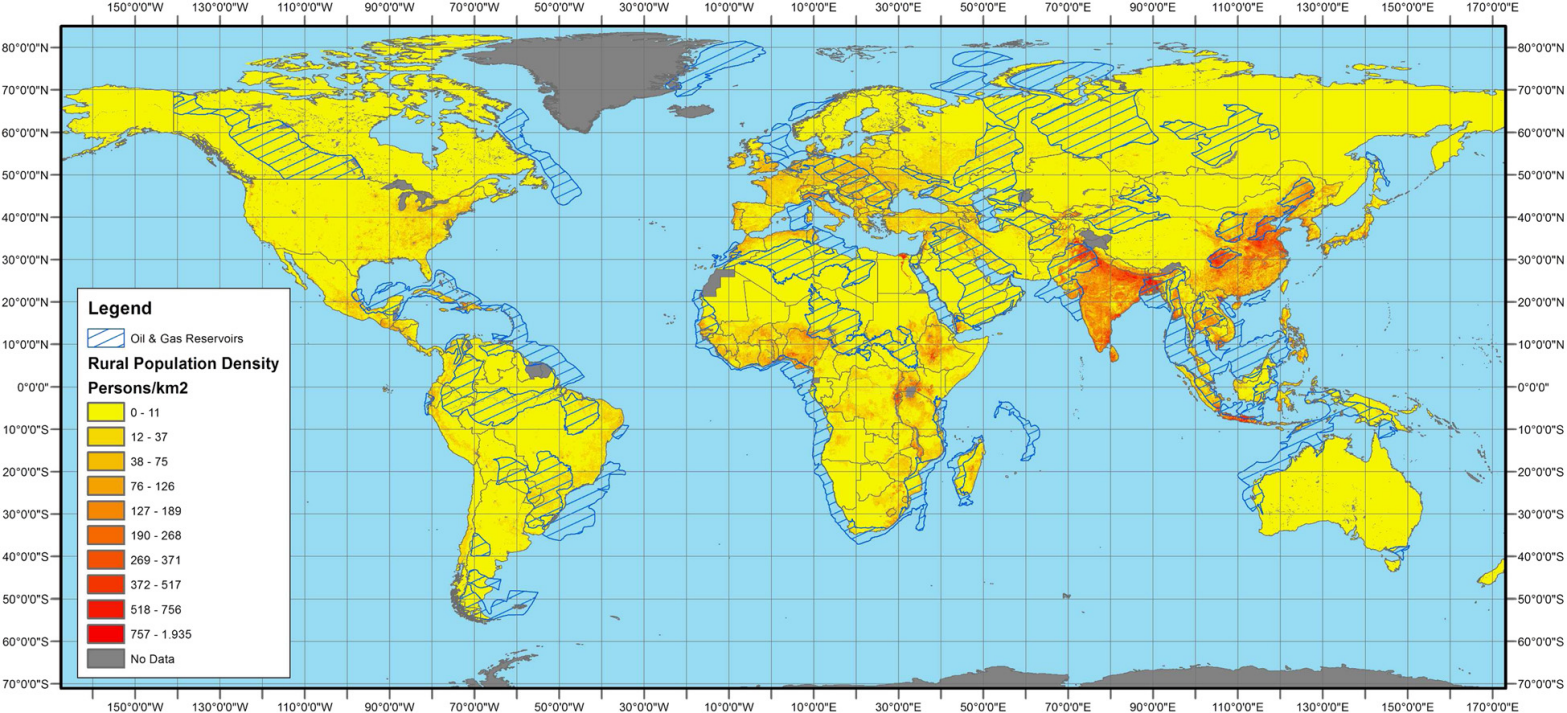
Map of rural population density and conventional oil and gas reserves. Conventional oil reservoirs and gas reservoirs based on data from the United States Geological Survey [27]. Rural population density based on the Food and Agricultural Organization (FAO) estimates [4]

Oil industry includes search of oil fields and extraction of crude oil to the surface, transport and storage of crude oil or refined petroleum products and the refinery and process of crude oil. Each of these phases lead to different exposures for human populations. There are no solid data on the overall population living close to oil facilities. We estimated that 638 million persons in LMICs inhabit rural areas close to conventional oil reservoirs (Fig. 1). We estimated this figure by overlapping conventional oil reservoirs (based on data from the United States Geological Survey and following the methodology used by Butt et al. [3]) and maps of rural population density [4]. We used oil reservoirs instead of current areas of extraction (i.e. oil blocks) due to lack of publicly available data.

The most common acute effects reported after exposure to oil spills among cleanup workers are respiratory, eye and skin symptoms, headache, nausea, dizziness and fatigue. Chronic effects include psychological disorders, lower respiratory tract symptoms and reduction of lung function. Genotoxicity and alterations in hormonal status have also been described [2]. High levels of aluminium, nickel, lead and zinc have been reported in volunteers and workers involved in cleaning up activities after the Prestige oil spill [5].

The health effects among people residentially exposed to oil extraction related contamination have been poorly studied. Evaluating these populations is important for several reasons: First, there are large populations living close to oil fields and persons are exposed during long periods of time. Most of the active oil fields are 50-years old, but could be active for longer [6]. Second, routes of exposure differ between workers and people living close to oil fields, who can be exposed by ingestion of contaminated waters/foods and by dermal contact with contaminated water and/or land during daily activities (e.g. bathing, agricultural activities, etc.). Third, individuals potentially more susceptible to the effect of contamination and not normally occupationally exposed, such as infants, children, pregnant women, elderly and people with previous health conditions, are also exposed.

There are 11 studies examining potential health effects of exposed communities. Ten of these studies have been conducted in the Ecuadorian and Peruvian Amazon [7–16] and one in the Niger Delta [17]. There are no health studies in other LMICs involving oil-extraction facilities. The study from the Niger Delta, reports higher frequency of neurological, haematological and irritation symptoms in inhabitants from a community were the main source of drinking water is contaminated with refined oil products, compared to a neighbouring community [17]. Some of the studies from the Ecuadorian Amazon reported higher risk symptoms previously described among cleanup workers after oil spills, such as fatigue, respiratory and eyes irritation and headaches [7], and higher risk spontaneous abortions [9] among women from exposed communities. Studies from the Peruvian Amazon compared blood lead levels among indigenous children and adults according to distance from place of residence to oil fields [14, 15]. Blood lead levels were high in the area, but no association was detected between blood lead levels and distance to oil extraction sites. Evidence on cancer risk is contradicting. Three studies conducted in the Ecuadorian Amazon identified increased cancer risk [8, 10, 11] in exposed areas. However a re-analysis of one of this studies [8] conducted by researchers funded by oil companies did not identify an increased risk [12]. Studies on cancer mortality were also conducted by researchers funded by oil companies and did not observe increased cancer mortality in the area [13, 16]. All these cancer studies shared methodological limitations such as potential errors in population estimates, no information on length of residence in the county, lack of information on occupational exposures and other important confounders. Genotoxiticy, which is directly associated with cancer risk, has been consistently observed in people exposed to oil spills [2].

Oil extraction related contamination leads to exposure to a mixture of contaminants. Produced waters originate in the natural oil reservoir and are separated from oil and gas in the production facility. Produced waters represent the major petroleum–derived waste [18]. They contain toxic compounds of natural origin, such as polycyclic aromatic hydrocarbons (PAHs), BTEX (benzene, toluene, ethylbenzene, and xylenes), heavy metals and occasionally naturally occurring radioactive materials, and may also contain chemicals from drilling fluids and treatment chemicals [18]. Exposure to produced water has been mainly studied among aquatic fauna in offshore production water, and negative effects on development, growth and immune response amongst others have been reported [19]. In onshore operations, production water should be re-injected to wells. Ninety-two percent of barrels of produced water generated in 1995 in US onshore production activities were re-injected [20]; however, dumping produced waters into rivers and streams has been common practice in a number of countries until recently [21, 22]. Natural gas flaring is also a common practice in oil fields. It leads to exposure to volatile organic compounds (VOCs), nitrogen dioxide (NO_2_), sulphur dioxide (SO_2_), PAHs and benzo[a]pyrene [23].

In remote areas of LMICs environmental legislation is less restrictive and control by supervisory bodies may be lacking [21]. The activity of Chevron-Texaco in the Ecuadorian Amazon is a fair example. Chevron-Texaco operated in the Ecuadorian Amazon between 1964 and 1992. In 2013, after 22 years of legal proceedings, the Ecuadorian Supreme Court ruled that USD 9.5 billion should be awarded to the plaintiffs (i.e. 30,000 mestizo and indigenous peoples) by Chevron-Texaco. This sentence was based on damage to the human health, water supply, and ecology among other harms [24]. In the Peruvian Amazon, there is lack of technical legislation on permissible levels of many pollutants on the practices for the management of production waters, drillings muds and gas flaring. The activities of oil companies operating in the area have led to contamination of air, water and soils in residential areas close to oil fields [25].

## Conclusions

Large populations are potentially exposed to oil extraction related contamination through residence in areas where oil extraction is conducted, especially in LMICs. Adverse health effects of exposure to oil extraction related compounds are known among oil industry workers and oil spills cleanup workers [2], but there are surprisingly few studies focusing on populations residentially exposed. There is a need for research through the conduct of methodologically robust studies in exposed populations worldwide, as also recommended by a United Nations Environment Programme (UNEP) report [26]. Such studies should include individual exposure assessment. There is currently enough technology available to allow collection of biological samples in remote areas and transportation to laboratories (e.g. sun-powered freezers). Therefore, measurement of biomarkers of exposure and effect (e.g. level of metals in blood/urine, lead isotopic ratios to trace sources, measurement of 1-hydroxypyrene in urine, presence of PAHs DNA adducts, evaluation of chromosomal damage by comet assay or micronucleus test, amongst others) should be included in future studies. These studies should be promoted to understand the health risks associated with residential exposure to oil related contamination, support effective control policies to avoid such contamination and sustain public health recommendations and policies to avoid exposure in already contaminated places.

## Abbreviations

BTEX: benzene, toluene, ethylbenzene, and xylenes
FAO: food and agricultural organization
LMICs: low and middle income countries
NO_2_: nitrogen dioxide
PAHs: polycyclic aromatic hydrocarbons
SO_2_: sulphur dioxide
UNEP: United Nations Environment Programme
VOCs: volatile organic compounds
